# Brain-derived neurotrophic factor serum levels as a candidate biomarker for withdrawal in crack heroin dependence

**DOI:** 10.1186/s13011-024-00591-0

**Published:** 2024-01-20

**Authors:** Enam Alhagh Charkhat Gorgich, Mohammad Gol Rigi, Hamed Fanaei, Houman Parsaei, Abdolhakim Ghanbarzehi

**Affiliations:** 1 Department of Anatomy, School of Medicine, Iranshahr University of Medical Sciences, Iranshahr, Iran; 2 Department of Law, Hatef Higher Education Institute, Zahedan, Iran; 3 Pregnancy Health Research Center, Zahedan University of Medical Sciences, Zahedan, Iran; 4 Department of Physiology, School of Medicine, Zahedan University of Medical Sciences, Zahedan, Iran; 5 Nervous System Stem Cells Research Center, Semnan University of Medical Sciences, Semnan, Iran; 6 Department of Anatomy, School of Medicine, Semnan University of Medical Sciences, Semnan, Iran; 7 Student Research Committee, Iran University of Medical Sciences, Tehran, Iran; 8 Department of Neuroscience, School of Advanced Technologies in Medicine, Iran University of Medical Sciences, Tehran, Iran; 9 Department of Physiology, School of Medicine, Iranshahr University of Medical Sciences, Iranshahr, Iran

**Keywords:** Crack heroin, Brain-derived neurotrophic factor, Withdrawal, Neuroplasticity, Opiate dependence, Biomarkers

## Abstract

**Background:**

Crack heroin is a novel opiate derivative with highly addictive properties and unfamiliar health consequences. It causes a variety of brain dysfunctions that are mediated by neurochemical alterations and abnormal neuroplasticity. Brain-derived neurotrophic factor (BDNF) is a widely recognized biological marker implicated in the neuropathology of substance use during substance use disorder and withdrawal. Its involvement can significantly contribute to the severity of withdrawal symptoms. Hence, this study aimed to evaluate BDNF levels in crack heroin users before and after withdrawal.

**Methods:**

In this cross-sectional study, 148 male participants were recruited and divided into two groups: persons with crack heroin use disorder (*n* = 74) and the controls (*n* = 74). The BDNF serum levels were measured in both crack heroin users and control groups upon hospitalization and again after twenty-one days of withdrawal using the enzyme-linked immunosorbent assay.

**Results:**

The results demonstrated that BDNF levels in persons with crack heroin use disorder upon admission were significantly lower than the levels observed upon discharge and in the control group (*p* < 0.05). Additionally, a significant difference in BDNF levels was found between persons with crack heroin use disorder at admission and discharge (*p* = 0.038). Furthermore, BDNF levels showed an inverse correlation with the daily dose of substance use (*r*= -0.420, *p* = 0.03) and the duration of crack heroin use (*r*= -0.235, *p* = 0.001).

**Conclusions:**

A progressive increment in BDNF levels during early detoxification is associated with the daily amount of substance use and the duration of substance use. Our findings suggest that changes in BDNF serum levels during crack heroin use disorder and withdrawal could serve as potential biomarkers for assessing the intensity of withdrawal symptoms and substance use-related behaviors.

## Background

Substance use is recognized as a major multifaceted and relapsing public health problem worldwide, with significant socio-economic consequences [1]. Crack cocaine is a brain-stimulant narcotic substance with a chemical composition and derivation similar to cocaine; however, its production method is different, making it highly addictive compared to cocaine [2, 3]. For the first time, crack cocaine emerged in Europe and the United States in the late 1970s, and since then, its use has become increasingly prevalent in other societies [4]. The use of crack cocaine has significant consequences on the brain.

When crack-cocaine is ingested, it can inhibit the reuptake and recycling of catecholamines, particularly dopamine and serotonin for example, as soon as is taking crack-cocaine it can inhibit the reuptake and recycling of catecholamines, particularly dopamine, and serotonin. This leads to the accumulation of excess levels of these neurotransmitters in the brain circuits associated with pleasure, reward, and substance use centers [5, 6]. Consequently, it results in feelings of euphoria, alertness, and heightened energy levels [7]. Moreover, research has demonstrated that even short-term consumption of crack cocaine can lead to the rapid development of intensity and obsession with substance use disorder. This, combined with the high potential for relapsing, makes crack cocaine use one of the most dangerous consequences [7, 8].

In recent years, the use of a new form of heroin-based opiate (nicknamed “crack”) with different symptoms has rapidly spread as a highly addictive substance in eastern societies, especially in Iran (known as Iranian crack) [9]. This substance, a condensed and pure form of heroin, causes short-term euphoria, which is considered the major motivation behind its use. However, it is also accompanied by unpleasant side effects, such as sleepiness, miosis, rhinorrhea, epiphora, pain, slowed functioning, and loss of consciousness. Furthermore, crack is associated with various physiological, behavioral, and cognitive impairments [10, 11]. Crack heroin, due to its quick and easy preparation, easy access, and odorless nature, has a high prevalence. According to unofficial reports, crack usage is second only to opioid use in terms of frequency among persons who use substances in Iran [9].

Moreover, its pharmacokinetic properties lead to rapid absorption and delivery in the central nervous system (CNS). Consequently, this can facilitate the development of severe substance use patterns and withdrawal symptoms after a short period of substance use [8, 12]. Studies have demonstrated that crack heroin has a distinct chemical composition, substance use pattern, side effects, and clinical symptoms compared to common crack [9, 10, 13].

A wealth of evidence suggests that substance use leads to alterations in the immunological and hormonal equilibrium state of the brain to adapt to the new molecular and neurochemical status caused by substance use disorder. These alterations result in structural, functional, and long-term neuroplasticity changes in the neuronal circuits of individuals with substance use [14–16]. Following these new adaptations in neuronal circuits, changes also occur in neurobiological biomarkers. Importantly, some of these biomarkers can be used to predict the severity of the disease, treatment outcome, craving, relapse, and the intensity of crack withdrawal symptoms [16–18].

In this regard, brain-derived neurotrophic factor (BDNF) is the best-known and most abundant neurotrophin in the brain. As a consequence of addictive substance use, it leads to structural alterations in neural circuits, particularly in the brain areas associated with the reward circuitry in substance use disorder [11, 19]. BDNF participates in several essential functions that are necessary to maintain the brain’s equilibrium state, including neuron survival, neurogenesis, neurotransmitter modulation, and, notably, synaptic adaptations and neuroplasticity changes [19].

During substance use or withdrawal stages, BDNF serum levels undergo significant fluctuations related to substance use, and this is involved in many substance-related behavioral changes, including relapse, substance craving, and sensitization [11, 20, 21]. As a result, measuring BDNF serum levels can be a valuable strategy for predicting relapse, craving, and withdrawal severity at different stages, as well as assessing treatment adherence in individuals with substance use disorder [21–23]. Sordi et al. revealed that BDNF levels at admission and discharge were lower than those in the control group during early abstinence from crack cocaine, although an increment was found in BDNF after a withdrawal period. Their findings imply that variations in BDNF levels could serve as potential biomarkers for assessing substance use severity [23]. Pianca et al. also observed a reduction of BDNF levels in adolescent crack cocaine users before and after abstinence compared to the controls. They highlighted that these levels were associated with recent substance use, indicating their potential as biomarkers for assessing the progression and prognosis of substance use [7]. In another investigation by Zhang et al. concerning BDNF serum levels in opiate users, it was found that serum levels of BDNF were increased during early heroin withdrawal. These findings suggest that BDNF levels may play a key role in opiate-induced substance use disorder and withdrawal [24]. Since different substances can cause varying substance use patterns in vulnerable individuals, they also have different effects on distinct components of the substance use cycle [25]. Therefore, studying biomarkers that can reflect neurochemical changes in the brain during substance use and withdrawal periods can be crucial for clinical use. These biomarkers can serve as a basis for proposing the disease course and assessing the severity of abstinence symptoms in persons with a substance use disorder undergoing rehabilitation.

At the present time, little is known about changes in BDNF serum levels in crack heroin users. Thus, the main goal of the current study was to evaluate BDNF serum levels in persons with crack heroin use disorder before and after withdrawal.

## Methods

### Study design

The present case-control study investigated BDNF serum concentrations to elucidate the underlying mechanisms and identify neurobiological molecules that could potentially serve as biomarkers in persons with crack heroin use disorder during substance use and withdrawal periods, as compared to persons without substance use disorders.

### Participants

A total of 148 male participants were investigated and divided into two independent groups: (I) Persons with crack heroin use disorder and (II) persons without substance use disorder as the controls.

The 74 persons with crack heroin use disorder were recruited after fulfilling the Diagnostic and Statistical Manual of Mental Disorders, Fifth Edition, (DSM-5) criteria, as determined through clinical examination by two expert psychiatrists specializing in opiate (heroin) use disorder. The persons with crack heroin use disorder were referred to Ofough Bidari and Bahari Digar substance use treatment centers in Zahedan, Iran. The persons with crack heroin use disorder provided consent to participate in and complete the substance abstinence/detoxification program (21 days) during hospitalization (Inpatient unit). During the hospitalization period, persons with substance use do not receive any treatment/medication.

The inclusion criteria for the persons with crack heroin use disorder were as follows:

 [1] age between 18 and 60 years old, [2] a history of at least one year of persistent crack heroin use, [3] a positive urine test for opiates on admission, and [4] no prior treatment or abstinence-related to substance use disorder during the past year. Persons with substance use disorder with the following conditions were excluded from the study: [1] any neurobiological, neurodegenerative, and psychiatric disorders, [2] a history of brain surgery and metabolic and chronic diseases, [3] infectious diseases, [4] HIV seropositivity, [5] acute withdrawal symptoms, [6] any substance use other than crack heroin or nicotine according to fulfilling the DSM-5 criteria, which was evaluated and ruled out by two experienced psychiatrists, [7] persons with mixed substance use [8] debilitating cognitive changes, and [9] cancer.

The 74 persons without substance use disorder were randomly selected from among volunteers with no history of substance use disorder and no diagnosis of neurological and psychological diseases. They had no family relationship with persons with crack heroin use disorder, no specific medication history, and no chronic metabolic diseases, as confirmed through medical history and physical examination conducted by the same psychiatrists.

### Sociodemographic characteristics

Sociodemographic information was gathered through standardized instruction during a structured interview as part of the precise clinical assessment. After ensuring that the participants met the inclusion and exclusion criteria, their information was recorded.

### Blood sampling and processing

A peripheral blood sample (10 ml) was collected from the brachial vein of all participants after an 8-hour fasting state. The blood was transferred into a tube without any anticoagulant. In the crack heroin group, blood sampling was conducted at two times: (I) at admission as the baseline within the first 24 h of registration, and (II) at the end of the treatment period on the 21st day of withdrawal, following a negative urine opiate test. For participants in the control group, blood sampling was performed only once on the evaluation day.

As soon as the blood was collected, serum was prepared by centrifugation at 4000×g for 10 min at 4 °C. The resulting supernatant was then stored at -80 °C before the immunoassay.

### The BDNF serum levels measurement

Serum BDNF levels in the crack heroin group were determined at baseline and after three weeks of follow-up and compared to the controls using a commercially available enzyme-linked immunosorbent [18] assay kit as per the manufacturer’s protocol (R&D Systems, Minneapolis, Minnesota, MN, USA).

Briefly, flat-bottomed 96-well microtiter plates were coated with 4 µg/ml of anti-BDNF capture antibody in PBS overnight at 4 °C. The plates were then rinsed twice with wash buffer and incubated with the samples diluted 1:200 in 1% bovine serum albumin. The BDNF standard curve ranged from 7.8 to 500 pg/ml. After washing the plates, 0.2 µg/ml of the anti-BDNF detection antibody was added and incubated for 2 h at room temperature. The samples were rinsed again and incubated with streptavidin-peroxidase conjugate in sample diluent (diluted 1:200) for 20 min. After rinsing, the samples were incubated with substrate solution and then with sulfuric acid (H_2_SO_4_) as the stop solution. Subsequently, the BDNF concentrations were calculated spectrophotometrically by measuring the absorbance at 450 nm wavelength using a microplate reader. The proceedings were conducted in duplicate by an expert operator who was blinded to the samples and the study design. The concentrations were represented as pg/mL. The intra-assay and inter-assay coefficients of variation were both less than 10%.

### Statistical analysis

The distribution of all variables was assessed using the Shapiro-Wilk test. The findings were presented as mean ± standard error of the mean (SEM). The independent Student’s t-test was applied to determine the difference in BDNF levels between the two groups. To compare the levels of BDNF at admission and discharge times, the paired t-test was used. Pearson’s correlation coefficient was used to assess the correlation between BDNF and substance-related characteristics. Data analysis and graph representations were performed using SPSS version 22.0 and GraphPad Prism 8.0 software for Windows, respectively. The significance level was set at *p* < 0.05.

## Results

### Sociodemographic characteristics of participants

In the present study, 74 persons with crack heroin use disorder were included in the final analysis. But in total, 117 individuals were admitted to the substance use treatment centers with the diagnosis of crack heroin use disorder. During the abstinence period, there were 43 individuals for various reasons, such as leaving the abstinence period before the end of the period (*n* = 27), severe insomnia disorders requiring taking medication (*n* = 4), severe anxiety and mood disorders (*n* = 5), the occurrence of severe withdrawal symptoms (*n* = 7) as diagnosed by the psychiatrists were excluded from the study. During the initial screening of the study, individuals with a history of mixed substance use were excluded from the study by psychiatrists.

We assessed a total of eighty volunteers, six of whom were excluded from the study because they had chronic metabolic disorders, heavy smoking, or were using a specific medication.

A total of 148 male participants, including 74 persons with crack heroin use disorder and 74 controls, were recruited for the present study. The mean age in persons with crack heroin use disorder and persons without substance use disorder was 31.09 ± 0.8 and 32.47 ± 1.25 years, respectively. The sociodemographic characteristics of the persons with crack heroin use disorder and the controls are detailed in Table 1. As shown in Table 1, there were no significant differences in age, education years, marital status, and body mass index (BMI) between the two groups (*p* > 0.05). However, a significant difference was found in weight between persons with crack heroin use disorder and the controls (*p* < 0.05).

**Table 1:** Sociodemographic and substance use-related characteristics in persons with crack heroin use disorder and the controls

Characteristics		Crack heroin dependence (*n* = 74)	Controls (*n* = 74)	*p*-value
Age (years)		31.09 ± 0.8	32.47 ± 1.25	0.33
BMI		21.96 ± 2.45	23.54 ± 3.60	0.27
Education (years)		7.15 ± 0.67	8.13 ± 9.46	0.79
Marital status	Single	32	16	0.52
	Married	40	48	
Age of the first substance use		20.8 ± 3.20	-	NA
Duration of substance use (years)		4.75 ± 3.62	-	NA
Daily dose of substance use (gr)		1.36 ± 0.5	-	NA

* Significant, NA: Not applicable, BMI: Body mass index

### The BDNF serum levels

**Fig. 1:**
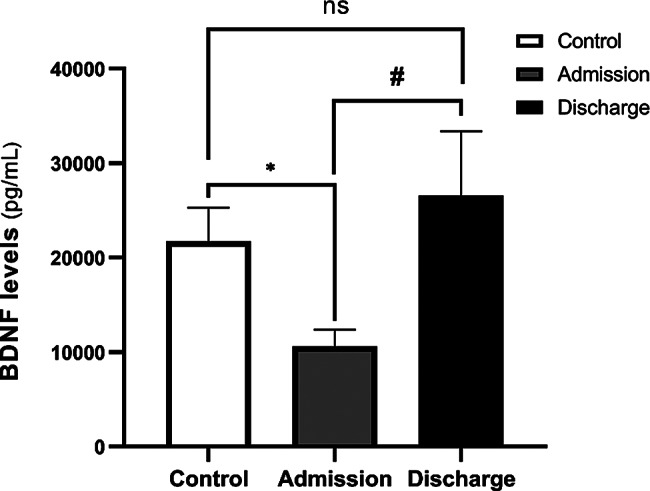
Evaluation of serum BDNF levels in the controls and persons with crack heroin use disorder on admission and discharge. Data are expressed as mean ± SEM. Differences between the controls and persons with crack heroin use disorder by the independent t-test and between persons with crack heroin use disorder after and before detoxification were analyzed via the paired t-test. (*p < 0.01 indicates significant difference between the controls and persons with crack heroin use disorder on admission; **#***p* < 0.05 indicates significant difference between serum BDNF levels on admission and discharge; ns *p* > 0.05 no significant differences were found between serum BDNF levels in persons with crack heroin use disorder on discharge and the controls)

The results revealed a significant difference between BDNF levels in persons with crack heroin use disorder at baseline (10,610 ± 1803 pg/mL) and the controls (21,717 ± 3591 pg/mL) (*p* = 0.008). However, there was no statistically significant difference in BDNF levels between persons with crack heroin use disorder on discharge (26,555 ± 6840 pg/mL) and the controls (21,717 ± 3591 pg/mL) (*p* = 0.52) (Fig. 1). Furthermore, a significant difference was observed between BDNF serum levels of persons with crack heroin use disorder at baseline and post-detoxification (*p* = 0.038) (Table 2).

**Table 2:** The BDNF serum level values in persons with crack heroin use disorder and the controls

Test	Time	Crack heroin user disorder (*n* = 74)	Controls (*n* = 74)	*p*-value
BDNF (pg/mL)	Admission	10,610 ± 1,803	21,717 ± 3,591	0.008*
	Discharge	26,555 ± 6,840	21,717 ± 3,591	0.52

* Significant

In other words, the serum BDNF levels in persons with crack heroin use disorder at baseline were significantly lower compared to levels after withdrawal/abstinence and compared to the controls. The results also indicated that despite the increase in serum BDNF levels at the end of the 21-day withdrawal period, a significant difference was not observed in relation to the control levels.

### Association between BDNF levels, demographic and substance-related characteristics

The results indicated that BDNF serum levels in persons with crack heroin use disorder were not significantly associated with age, BMI, education, marital status, and age of first substance use (*p* > 0.05). However, serum BDNF levels were negatively correlated with the duration of crack heroin use disorder (*r* = -0.235, *p* = 0.001) and the daily amount of its use (*r*= -0.420, *p* = 0.03).

## Discussion

The findings of the current study demonstrated a significant increase in serum BDNF levels after the 21-day inpatient withdrawal period of persons with crack heroin use disorder, as compared to the admission time. Additionally, we observed a rise in BDNF levels after hospital detoxification. To the best of our knowledge, this work represents the first report on BDNF serum levels in chronic crack heroin users at both admission and discharge times.

Substance use disorder is a chronic and intricate brain disorder accompanied by a broad spectrum of behavioral, cognitive, and neurochemical changes [26]. A growing body of evidence suggests that long-lasting substance use, especially psychostimulant substances, can lead to structural and functional changes in different brain regions, potentially resulting in transient or permanent maladaptive neuroadaptations [27, 28]. However, the underlying mechanisms of such changes and the neurobiology of opioid use disorder are still unknown. A better understanding of the mechanisms of substance-related neuroadaptations may help us to characterize the neurobiology of substance use disorders, interindividual variability in substance use, and treatment response [28].

Evidence obtained from various preclinical and clinical studies has demonstrated that neurotrophins, particularly BDNF levels, play an inseparable role in neuroadaptations induced by substance use and substance-related behaviors, such as craving, withdrawal severity, and relapse [8, 23, 29]. BDNF is a well-known neuropeptide that not only plays a role in neurodevelopment but also has a significant impact on many vital functions of the mesolimbic dopaminergic system, especially in substance use disorder. It has been proposed that the rewarding properties of substances originate from dopamine release in the ventral tegmental area (VTA) and projections to other related neuronal circuits [30].

It has been shown that BDNF, in addition to being involved in new neuroplastic and neuroadaptive modifications associated with substance use, also supports and maintains dopaminergic neurons in the midbrain and is involved in the modulation of dopamine release, contributing to the development of psychological dependencies in persons with opioid use disorder [31, 32].

A large body of evidence has demonstrated that the use of cocaine and heroin exerts their effects by disrupting the function of dopaminergic neurons located in the VTA and nucleus accumbens [30]. These pieces of evidence implicate that substance use leads to considerable neurotoxicity, resulting in structural and functional changes in different brain areas [15, 33]. Moreover, recent studies have shown that neurotrophin levels, especially BDNF, undergo changes during active substance use or post-detoxification of psychostimulants [22–24, 34]. Various studies on the effects of substance use on BDNF levels during substance use or withdrawal periods have reported heterogeneous and conflicting results.

Hirsch et al. reported a great variability in BDNF levels during the detoxification period in crack users. They also stated that plasma BDNF levels significantly decreased after a short 14-day detoxification period compared to the admission time [18]. On the contrary, in line with our findings, Pianca et al. revealed that BDNF serum levels in crack-cocaine users at admission time were significantly lower than at discharge time and compared to the controls. Their findings also showed an increase in BDNF levels after an early abstinence period (21 days) compared to the controls; however, this increment was not statistically significant [35]. Furthermore, Sordi et al. reported lower BDNF levels in persons with crack-cocaine use disorder at admission and discharge time compared to the control group. However, they observed that BDNF levels increased at discharge compared to hospitalization but not compared to the controls [23]. Our findings demonstrated that BDNF serum levels in persons with crack heroin use disorder at discharge were higher than at admission time and in the control group; however, these increased levels did not show a significant difference with the controls, which is consistent with other studies [23, 35, 36]. On the other hand, Zhang et al. found a significant increase in serum BDNF levels after 26 weeks of abstinence from heroin use when compared to the baseline. Their results revealed that alterations in BDNF levels during the protracted withdrawal period were correlated with withdrawal symptoms [37]. In this regard, clinical studies have shown that BDNF levels may increase during withdrawal to return to normal levels in order to restore normal functions in the damaged neuronal circuits caused by substance use [36]. In contrast to the above-mentioned studies, the findings of the case-control study conducted by Sarkar reported that BDNF serum levels did not differ between control subjects and opioid dependents. They also found similar findings regarding BDNF levels before and after 10 days of detoxification [38]. Based on the content, it appears that the neurotoxicity induced by substance use directly or indirectly affects the mechanisms of BDNF synthesis, leading to the suppression of its production. Evidence supporting this claim includes the decrease in BDNF levels following chronic substance use and the increase in BDNF levels during early abstinence periods [39]. Other evidence from clinical studies suggests that another possible reason for the neurobiological changes and increase in BDNF levels during early detoxification or withdrawal periods in substance users is likely due to the activation of compensatory mechanisms in the CNS in response to the neurotoxic effects of substance use [40, 41]. Such compensatory processes in substance users and other neurological disorders have been reported in various studies [15, 42–44]. It is possible that elevated peripheral BDNF levels are necessary to protect dopaminergic neurons from substance-induced neurotoxic effects. Meanwhile, it has been shown that BDNF plays a trophic role for midbrain dopaminergic neurons as it enhances their survival and protects them from neurodegenerative processes [45]. Reciprocally, it has been proposed that mesencephalic dopaminergic neurons are a key pathophysiological basis for substance use-related disorders. Among the neuroadaptive responses to addictive substances, structural and functional plasticity has received considerable interest. Both structural and functional neuroplasticity occur at pre-and post-synaptic sites in the mesolimbic dopaminergic axis [46, 47]. Additionally, studies have demonstrated that BDNF and extracellular dopamine act as two determining factors in neuronal remodeling and plasticity induced by substance use [46, 47]. Interestingly, these molecules share similar intracellular molecular pathways called MEK-ERK1/2 and PI3K-Akt-mTOR. These pathways selectively activate TrkB and dopamine D3 receptors, which play a role in perikaryon and dendrite growth, respectively [46]. This content indicates the close and mutual interaction of BDNF and dopamine in substance use-related disorders. Thus, evaluating BDNF serum levels in persons with crack heroin use disorder not only can be suggested as a valuable clinical biomarker to follow up the treatment process, predict substance-related behaviors, and determine withdrawal prognosis in the clinic, but it can also indirectly demonstrate changes that occur in the mesencephalic dopaminergic system.

Furthermore, considering the important role of BDNF as a valuable biomarker in substance use disorder, a recent study found that peripheral BDNF can indicate the severity of executive cognitive behaviors in persons with substance use disorder and may be used as a biomarker in these users [48]. Several studies have indicated a significant correlation between BDNF levels and the severity of substance use during the early stages of detoxification [23, 36]. Another study showed a negative association between changes in BDNF serum levels and long-term withdrawal symptoms [37]. Roso et al. reported that chronic use of cocaine is related to BDNF reduction, but increasing levels of BDNF during withdrawal are associated with cocaine craving and withdrawal symptoms [39]. Another study showed that high levels of BDNF after 3 weeks of cocaine withdrawal predicted earlier and more severe relapse [40]. Similarly, our findings also demonstrated an inverse correlation between the duration of crack heroin use, the daily amount of crack heroin use, and serum levels of BDNF after 21 days of detoxification in persons with a substance use disorder.

Based on these findings, it is possible that changes in BDNF levels after the detoxification period can serve as a potential biomarker for predicting substance-related behaviors, such as substance-seeking behaviors, relapse, and the severity of withdrawal symptoms.

There are a number of limitations in the present investigation. First, we only evaluated BDNF values in the serum of the participants and not in the brain. At this point, serum and peripheral BDNF levels may display some variation compared to the brain BDNF levels. Second, all the participants in our study were male, which limits the generalization and interpretation of our findings. In addition, the second evaluation of the BDNF level has been carried out after twenty-one days and it may not reflect variations over the longer term. In order to clarify the association between serum BDNF levels and the duration of abstinence, it will be necessary to long-term follow-up period. Another important limitation of the study was that neuropsychological tests and clinical evaluations related to craving and abstinence symptoms were not performed, which may be potentially associated with cognitive dysfunctions, neuroplasticity, and BDNF levels in crack heroin users. They need further investigation.

## Conclusion

Based on the findings, it can be concluded that the neurotoxicity induced by crack heroin suppresses BDNF, and a 21-day abstinence period from crack heroin use increases the serum levels of BDNF in crack heroin users. These changes in BDNF levels are associated with substance-related characteristics and may predict the intensity of withdrawal symptoms and substance-related behaviors. Therefore, alterations in BDNF levels during the substance use and withdrawal cycle can be utilized as a potential prognostic biomarker in the clinical setting to manage and predict substance use-associated behaviors caused by substance-related neurochemical changes. It seems that an increase in BDNF serum levels during the early abstinence period may be associated with crack heroin pathophysiology.

Given the intimate interaction of BDNF levels and the function of dopaminergic neurons, along with the vital role of dopamine in substance use, further studies to investigate and elucidate their relationship are highly recommended.

## Data Availability

Not applicable.

## Abbreviations

BDNF: Brain-derived neurotrophic factor
CNS: Central nervous system
DSM-5: Diagnostic and Statistical Manual of Mental Disorders, Fifth Edition
VTA: Ventral tegmental area
SEM: Standard error of the mean
